# Mucormycosis in adult hematopoietic stem cell transplant recipients during the antifungal prophylaxis era: a single-center retrospective case series

**DOI:** 10.1186/s12879-026-14191-9

**Published:** 2026-08-07

**Authors:** Aryan Shiari, Kelly Pennington, Hemang Yadav

**Affiliations:** 1 Division of Pulmonary, Critical Care, and Sleep Medicine, Mayo Clinic Health System, Eau Claire, WI USA; 2 Division of Pulmonary, Critical Care, and Sleep Medicine, Mayo Clinic, Rochester, MN USA

**Keywords:** Mucormycosis, Mucorales, Hematopoietic stem cell transplantation, Hematopoietic stem cell transplant recipients, Antifungal prophylaxis, Breakthrough mucormycosis, Therapeutic drug monitoring, Graft-versus-host disease, Invasive fungal infection

## Abstract

**Background:**

Mucormycosis is an uncommon but severe invasive fungal infection in hematopoietic stem cell transplant recipients. Contemporary cases may occur outside the classic early neutropenic post-transplant window and during antifungal prophylaxis.

**Methods:**

We performed an International Classification of Diseases code-screened retrospective case series using the institutional denominator of adult hematopoietic stem cell transplant recipients at Mayo Clinic from January 1, 2008, through December 1, 2025. Cases were adjudicated using 2020 European Organization for Research and Treatment of Cancer and Mycoses Study Group Education and Research Consortium criteria. We analyzed transplant characteristics, immune and pulmonary host factors, prophylaxis and therapeutic drug monitoring, diagnostic evidence, management, critical care use, and outcomes.

**Results:**

Among 6,340 adult transplant recipients, 13 met criteria for invasive mucormycosis; eleven had allogeneic transplant and 2 had autologous transplant. Median age was 61 years, and median onset was 681 days after transplant. Underlying conditions included acute myeloid leukemia in 4 patients, chronic lymphocytic leukemia and chronic myeloid leukemia in 2 each, and diffuse large B-cell lymphoma, T-cell lymphoma, acute lymphoblastic leukemia, multiple myeloma, and VEXAS syndrome in one each. All patients were receiving systemic antifungal prophylaxis; 11 were receiving mold-active azoles and 6 were receiving posaconazole. Five patients had pre-diagnosis azole levels available. Neutropenia was present in 4 patients, while lymphopenia, low helper T-cell counts when measured, graft-versus-host disease, targeted or immunomodulatory therapies, and pulmonary host-defense abnormalities were frequent. Pulmonary or mixed pulmonary involvement occurred in 8 patients, disseminated disease in 3, and 9 cases were proven. Liposomal amphotericin B was used in 12 patients, source-control surgery in 6, intensive care unit admission in 6, mechanical ventilation in 5, and all-cause mortality was 5 of 13 at 30, 42, and 90 days.

**Conclusions:**

This series describes mucormycosis in adult transplant recipients rather than establishing causal attribution to transplantation in every case. The findings support diagnostic vigilance for Mucorales infection in chronically immunocompromised transplant recipients, including those remote from transplant, receiving prophylaxis, and without neutropenia.

**Supplementary Information:**

The online version contains supplementary material available at https://doi.org/10.1186/s12879-026-14191-9.

## Background

Mucormycosis is an invasive fungal disease caused by molds in the order Mucorales, a heterogeneous group that includes genera such as Rhizopus, Mucor, Rhizomucor, Lichtheimia, and Cunninghamella [1, 2]. Disease is uncommon but frequently severe in immunocompromised hosts, including hematopoietic stem cell transplant (HSCT) recipients, and may involve pulmonary, rhino-orbital-sinus, central nervous system, cutaneous, gastrointestinal, or disseminated sites [1–5].

Prior transplant and hematologic malignancy cohorts show that mucormycosis can occur during antifungal prophylaxis, including while receiving agents with variable or absent Mucorales activity [4, 6, 8, 11–16]. However, single-center patient-level descriptions remain useful because the bedside context varies across eras, prophylaxis strategies, diagnostic pathways, host immune phenotypes, and the availability of species-level identification or therapeutic drug monitoring (TDM). We therefore described invasive mucormycosis in adult HSCT recipients at our institution over a modern antifungal prophylaxis era, emphasizing timing, host factors, prophylaxis/TDM, diagnostic evidence, management, and short-term outcomes.

## Methods

### Study design and setting

We conducted a retrospective case series with an institutional adult HSCT denominator at Mayo Clinic, Rochester, Minnesota. The study period extended from January 1, 2008 through December 1, 2025. The Mayo Clinic Institutional Review Board reviewed the study and determined that it was exempt as retrospective minimal-risk research; the requirement for informed consent was waived. The study was conducted in accordance with the Declaration of Helsinki and applicable institutional policies.

### Case identification and definitions

The source population included 6,340 unique adult HSCT recipients. Potential cases were identified through ICD-9-CM and ICD-10-CM screening for mucormycosis or unspecified invasive mold infection codes, followed by detailed chart review. Proven and probable mucormycosis were adjudicated using 2020 European Organization for Research and Treatment of Cancer/Mycoses Study Group Education and Research Consortium criteria [7]. Disseminated mucormycosis was defined as involvement of at least 2 noncontiguous sites or sterile-site involvement with another organ site. A parallel microbiology or pathology text search was not performed.

### Variables and analysis

Study variables included transplant type, donor type, conditioning intensity, graft-versus-host disease (GVHD), neutropenia, absolute lymphocyte count (ALC), CD4 count, immunoglobulin G, corticosteroid dose, targeted or immunomodulatory therapies, antifungal prophylaxis, azole TDM, site of infection, diagnostic evidence, microbiology, antifungal therapy, source-control surgery, critical care use, and outcomes. Underlying hematologic disease was abstracted, but hematologic disease status at mucormycosis onset, the clinical indication for antifungal prophylaxis, diabetes, iron overload, amphotericin dose, end-of-treatment outcome, and in-hospital mortality were not uniformly captured in the prespecified abstraction dataset (Additional file 1: Table S2).

Mucorales-active prophylaxis was defined as posaconazole or isavuconazole prophylaxis; no patient was receiving isavuconazole prophylaxis at diagnosis. Because posaconazole activity varies across Mucorales genera and species, this category was interpreted as exposure to a Mucorales-active agent rather than as assured protection against every Mucorales organism [1, 16]. Analyses were descriptive. No comparative hypothesis testing or multivariable modeling was performed.Reporting followed the STROBE statement [10].

## Results

### Case identification and transplant context

**Fig. 1:**
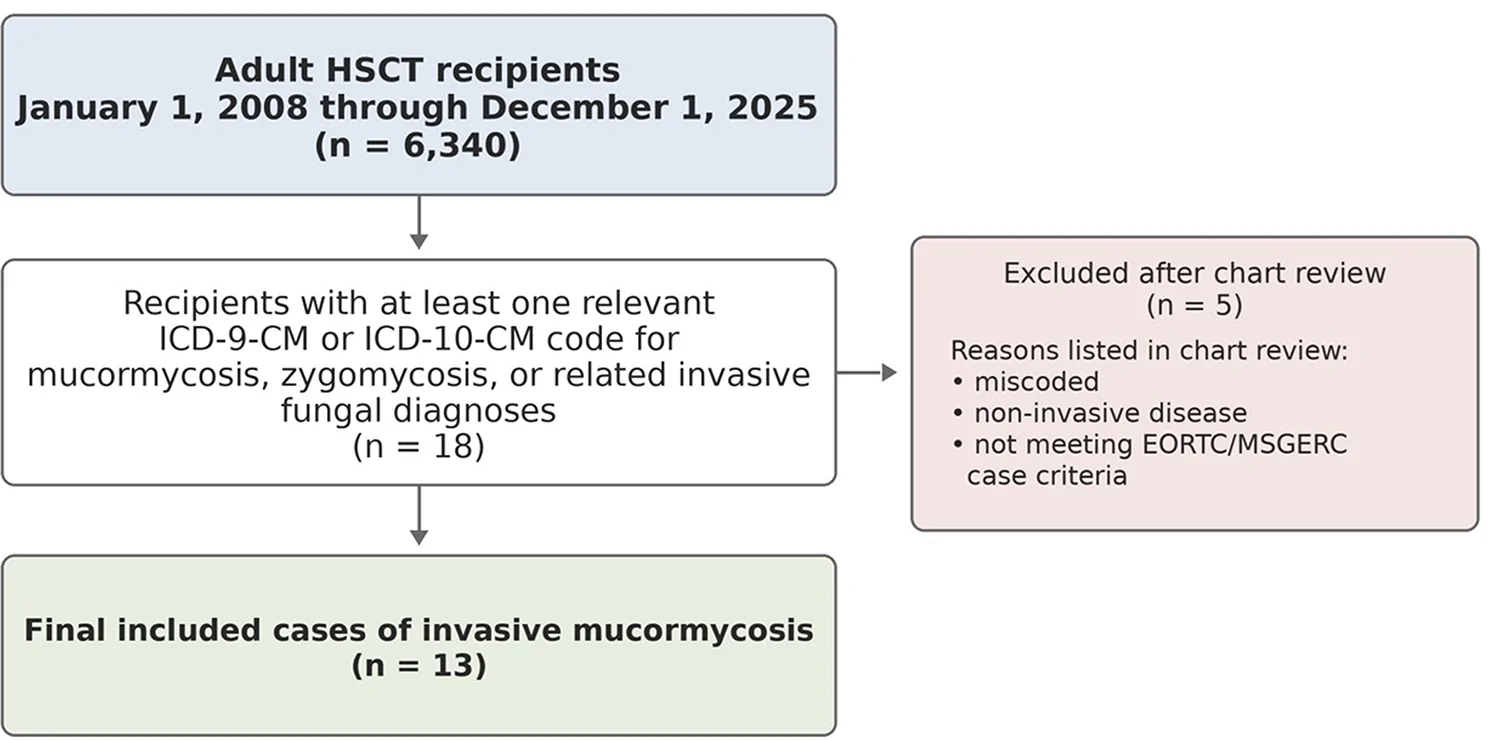
Flow diagram of ICD-screened case identification and chart-review adjudication. Abbreviations: EORTC/MSGERC, European Organization for Research and Treatment of Cancer/Mycoses Study Group Education and Research Consortium; HSCT, hematopoietic stem cell transplantation; ICD, International Classification of Diseases

Eighteen adult HSCT recipients had at least one relevant ICD code, and 13 met criteria for invasive mucormycosis, corresponding to a crude case proportion of 2.1 per 1,000 transplant recipients. The ICD-screened case-identification and chart-review adjudication process is shown in Fig. 1.

Eleven patients had allogeneic HSCT and two had autologous HSCT. The autologous HSCT recipients had diffuse large B-cell lymphoma and multiple myeloma. Underlying conditions across the cohort were acute myeloid leukemia in four patients, chronic lymphocytic leukemia in two, chronic myeloid leukemia in two, and diffuse large B-cell lymphoma, T-cell lymphoma, acute lymphoblastic leukemia, multiple myeloma, and VEXAS syndrome in one each. Median time from HSCT to mucormycosis onset was 681 days. Three cases occurred before day 200 after transplant (days 25, 41, and 192), whereas several remote cases occurred beyond 1 year. Table 1 summarizes key transplant, host, diagnostic, treatment, and outcome characteristics.

**Table 1:** Key characteristics and outcomes

Characteristic	Value
Adult HSCT denominator	6,340
Cases meeting criteria	13
Allogeneic/autologous HSCT	11/2
Underlying diseases	AML 4; CLL 2; CML 2; DLBCL, T-cell lymphoma, ALL, MM, and VEXAS syndrome one each
Median onset after HSCT	681 days (range 25-4063)
Cases before day 200 after HSCT	3
Systemic antifungal prophylaxis at diagnosis	13
Mold-active azole prophylaxis	11
Mucorales-active prophylaxis (posaconazole)	6
Pre-diagnosis azole TDM within 30 days	5
Pre-diagnosis TDM among posaconazole prophylaxis cases	4/6: 2 therapeutic, 1 subtherapeutic, 1 near prophylaxis threshold
Neutropenia at diagnosis	4
ALC < 500 cells/µL	7
CD4 < 200 cells/µL among available	5/7
Active GVHD	5
Any systemic corticosteroids at diagnosis	4
Other targeted/immunomodulatory therapy recorded	6
Pulmonary or mixed pulmonary involvement	8
Pulmonary host-defense/structural factor among pulmonary/mixed cases	6/8
Proven/probable mucormycosis	9/4
Species-level identification available	3
Other mold coinfection	3
Disseminated disease	3
Liposomal amphotericin B use	12
Definitive source-control surgery	6
ICU admission	6
Mechanical ventilation	5
30-day all-cause mortality	5
42-day all-cause mortality	5
Cumulative 90-day all-cause mortality	5

Abbreviations: ALC, absolute lymphocyte count; ALL, acute lymphoblastic leukemia; AML, acute myeloid leukemia; CLL, chronic lymphocytic leukemia; CML, chronic myeloid leukemia; DLBCL, diffuse large B-cell lymphoma; GVHD, graft-versus-host disease; HSCT, hematopoietic stem cell transplantation; ICU, intensive care unit; MM, multiple myeloma; TDM, therapeutic drug monitoring; VEXAS, vacuoles, E1 enzyme, X-linked, autoinflammatory, somatic. Values are counts unless shown as n/N proportions or median (range); percentages are not shown in this summary table

### Host factors, prophylaxis, and TDM

Active GVHD was present in five patients, and pulmonary GVHD or bronchiolitis obliterans syndrome was documented in four. Four patients were receiving systemic corticosteroids at diagnosis. Neutropenia was present in four patients, while ALC was below 500 cells/µL in seven. CD4 count was available in seven patients and was below 200 cells/µL in five. Six patients had other targeted or immunomodulatory therapy recorded, including epcoritamab, pembrolizumab, abatacept, venetoclax, daratumumab/pomalidomide/dexamethasone, rituximab, or donor lymphocyte infusion exposure (Additional file 1: Table S4).

All 13 patients were receiving systemic antifungal prophylaxis at diagnosis. Eleven were receiving mold-active azoles: posaconazole in six, voriconazole in four, and itraconazole in one; two were receiving fluconazole. Azole TDM was available in eleven patients overall, but only five had levels obtained within 30 days before diagnosis. Among the six patients receiving posaconazole prophylaxis, pre-diagnosis TDM was available in four: two levels were therapeutic, one was subtherapeutic, and one was near the prophylaxis threshold but below treatment-level exposure according to clinical documentation (Additional file 1: Table S6).

### Clinical syndrome, diagnosis, and microbiology

Primary sites were pulmonary in seven patients, rhino-orbital-sinus in three, mixed rhino-orbital-sinus plus pulmonary in one, central nervous system in one, and cutaneous in one. Disseminated disease occurred in three patients. Among eight patients with pulmonary or mixed pulmonary involvement, six had pulmonary GVHD/bronchiolitis obliterans syndrome, bronchiectasis, recurrent pulmonary infections, chronic aspiration risk, or another structural pulmonary abnormality.

Nine cases were proven and four were probable. Histopathologic invasion was documented in eight patients; sterile-site culture evidence was recorded in nine; bronchoalveolar lavage culture was positive in seven; sputum culture was positive in two; and molecular evidence was recorded in one. Case-level diagnostic pathways are shown in Additional file 1: Table S5. Species-level identification was available in three cases: Rhizomucor pusillus in two and Rhizopus oryzae in one. Other cases were reported at the genus or Mucorales level. Other mold coinfection was recorded in three patients, all involving Aspergillus species or complex.

### Management and outcomes

Liposomal amphotericin B was used in 12 patients; no amphotericin B deoxycholate or amphotericin B lipid complex use was recorded. Initial regimens included liposomal amphotericin B plus posaconazole in 8 patients, liposomal amphotericin B plus voriconazole in two, liposomal amphotericin B alone in two, and posaconazole monotherapy in one. Ten patients received active antifungal therapy within 24 h of diagnostic recognition. Amphotericin dose was not captured in the structured dataset.

Definitive source-control surgery was performed in 6 patients, including cases with pulmonary (*n* = 3), rhino-orbital-sinus (*n* = 2), and central nervous system (*n* = 1) primary involvement. Median time from diagnosis to source-control surgery among operated patients was 4.5 days (range 0–6). One additional patient underwent biopsy/debridement but definitive source control was not feasible. Case-level treatment details are provided in Additional file 1: Table S7.

ICU admission occurred in six patients and mechanical ventilation in five. All-cause mortality was 5 of 13 at 30 days, 42 days, and 90 days; all deaths occurred by day 30, with no additional deaths recorded between days 31 and 90 among 30-day survivors. Mortality was 3 of 3 among patients with disseminated disease and two of ten among those without dissemination; one of six among patients who underwent definitive source-control surgery and 4our of seven among those without definitive source control; and five of six among patients requiring ICU admission and zero of seven among those not requiring ICU admission (Additional file 1: Table S8).

## Discussion

This single-center series describes mucormycosis in adult HSCT recipients during an era of routine antifungal prophylaxis. The cohort remained small, but several findings are clinically relevant: many infections occurred outside the immediate peri-transplant period, all patients were receiving prophylaxis, neutropenia was absent in most cases, pulmonary host-defense abnormalities were common among pulmonary or mixed cases, and short-term mortality remained substantial.

The apparently late median onset requires cautious interpretation. Three cases occurred before day 200, but remote cases shifted the median to 681 days. Several explanations are possible, including prolonged survival with chronic immune dysfunction, chronic GVHD or pulmonary GVHD biology, ongoing pulmonary structural vulnerability, relapsed or refractory hematologic disease, later immunomodulatory or antineoplastic therapies, and referral patterns. Because disease status at mucormycosis onset and the indication for ongoing prophylaxis were not uniformly captured, these explanations remain hypotheses rather than conclusions. In addition, ICD-based screening could miss uncoded cases, including early cases in which mucormycosis was not coded or was not recognized; therefore, this series should not be interpreted as proving that early post-transplant mucormycosis was absent.

The prophylaxis findings also need nuance. Posaconazole was the only Mucorales-active prophylaxis agent used in the cohort, but posaconazole is not reliably active against every Mucorales genus or species, and breakthrough mucormycosis on Mucorales-active antifungals has been associated with poor outcomes in hematologic cancer cohorts [1, 16]. Voriconazole and fluconazole should not be interpreted as protective against Mucorales. Pre-diagnosis TDM was available in only a subset, limiting attribution of breakthrough disease to low exposure versus host or organism factors [6, 9].

Early active therapy may have contributed to the observed short-term outcomes. Ten of 13 patients received active therapy within 24 h of diagnostic recognition, and prior work in hematologic malignancy-associated zygomycosis has linked delayed amphotericin B-based therapy with increased mortality [17]. However, our study cannot establish causality because treatment timing, feasibility of surgery, ICU status, extent of disease, and underlying host trajectory were tightly interrelated. The descriptive subgroup outcomes suggest that dissemination, lack of feasible source control, and ICU requirement marked higher-risk presentations, but the numbers are too small for statistical inference.

This study has several limitations. The cohort was small, retrospective, and drawn from a single center, so estimates are imprecise and findings should be interpreted as descriptive. Case ascertainment relied on ICD screening followed by chart review, and no parallel microbiology/pathology text search was performed. Missed uncoded early cases are a plausible explanation for the late median onset, particularly if mucormycosis was not recognized or coded early after transplantation. Species-level identification was available for only 3 cases because routine clinical documentation did not consistently include ITS sequencing, MALDI-TOF, or reference laboratory species-level identification across the full study period. Diabetes, iron overload, hematologic disease status at mucormycosis onset, clinical indication for antifungal prophylaxis, timing/indication of targeted therapies, amphotericin dosing, end-of-treatment outcome, and in-hospital mortality were not uniformly available in the prespecified abstraction dataset and therefore were not analyzed. These omissions limit interpretation of late-onset disease and host-risk attribution.

## Conclusions

In this single-center series, mucormycosis among adult HSCT recipients often presented late, occurred during antifungal prophylaxis, and was not limited to neutropenic patients. The findings support careful attention to Mucorales infection in chronically immunocompromised transplant recipients with persistent hematologic, immune, or pulmonary vulnerability. Because late-onset cases may reflect later disease activity and other therapies rather than the original transplant alone, the results should be interpreted as a description of mucormycosis in HSCT recipients rather than causal attribution to transplantation in every case.

## Supplementary Information

Below is the link to the electronic supplementary material.

Supplementary Material 1

## Data Availability

Deidentified data underlying the reported results are available from the corresponding author upon reasonable request, subject to institutional approval and applicable privacy regulations.

## Abbreviations

ALC: Absolute lymphocyte count
BAL: Bronchoalveolar lavage
BOS: Bronchiolitis obliterans syndrome
GVHD: Graft-versus-host disease
HSCT: Hematopoietic stem cell transplantation
ICD: International Classification of Diseases
ICU: Intensive care unit
L-AmB: Liposomal amphotericin B
TDM: Therapeutic drug monitoring
