# Supplementary material for: Mucormycosis in adult hematopoietic stem cell transplant recipients during the antifungal prophylaxis era: a single-center retrospective case series
Source: BMC Infect Dis. 2026 Aug 7;26:1948. doi: 10.1186/s12879-026-14191-9 (PMC13737907; doi:10.1186/s12879-026-14191-9)
Supplement: Supplementary file 1 [file 12879_2026_14191_MOESM1_ESM.docx]

**Additional file 1. Supplementary Methods and Tables**

Mucormycosis in Adult Hematopoietic Stem Cell Transplant Recipients During the Antifungal Prophylaxis Era: A Single-Center Retrospective Case Series

Description: Supplementary Methods and Tables. This file contains denominator notes, exact confidence intervals, operational definitions, case-level host/prophylaxis/diagnostic/treatment details, subgroup outcome summaries, and a targeted structured literature comparison. This file is not a systematic review or meta-analysis.

**Supplementary Methods**

This supplementary file provides supporting methodological detail and deidentified descriptive data for the case series. Variable completeness notes are based on the prespecified retrospective abstraction dataset. When a diagnostic field reliably distinguished recorded negative results from not obtained or not recorded, test-performed denominators are shown. When a field did not reliably separate those categories, the variable is presented as a cohort-level evidence indicator rather than as a tested denominator.

A parallel Mayo Data Explorer microbiology/pathology search was not part of the prespecified analysis dataset. Such a search would query institutional microbiology, molecular, and pathology text fields for Mucorales-related terms and could identify non-ICD-coded cases. Because it was not performed, non-coded cases may have been missed.

Azole TDM interpretation was separated into pre-diagnosis and post-diagnosis measurements. The primary prophylaxis-exposure summary uses the closest azole level within 30 days before diagnosis. Post-diagnosis levels may reflect treatment changes rather than prophylaxis exposure.

**Supplementary Table S1. Exact 95% confidence intervals for selected descriptive estimates**

| **Estimate** | **n/N** | **Point estimate** | **Exact 95% CI** |
| --- | --- | --- | --- |
| Crude case proportion among adult HSCT recipients | 13/6340 | 2.1 per 1,000 | 1.1-3.5 per 1,000 |
| Systemic antifungal prophylaxis at diagnosis | 13/13 | 100% | 75.3-100% |
| Mold-active azole prophylaxis at diagnosis | 11/13 | 84.6% | 54.6-98.1% |
| Mucorales-active prophylaxis at diagnosis | 6/13 | 46.2% | 19.2-74.9% |
| Proven mucormycosis | 9/13 | 69.2% | 38.6-90.9% |
| Disseminated disease | 3/13 | 23.1% | 5.0-53.8% |
| Definitive source-control surgery | 6/13 | 46.2% | 19.2-74.9% |
| ICU admission within 14 days | 6/13 | 46.2% | 19.2-74.9% |
| Mechanical ventilation | 5/13 | 38.5% | 13.9-68.4% |
| 30-day all-cause mortality | 5/13 | 38.5% | 13.9-68.4% |
| 42-day all-cause mortality | 5/13 | 38.5% | 13.9-68.4% |
| Cumulative 90-day all-cause mortality | 5/13 | 38.5% | 13.9-68.4% |

**Supplementary Table S2. Data completeness and denominator notes for selected variables**

| **Variable** | **Interpretable cases** | **Notes** |
| --- | --- | --- |
| Underlying hematologic disease | 13/13 | No missing values in prespecified abstraction dataset. |
| Hematologic disease status at mucormycosis onset | 0/13 | Not uniformly abstracted in the prespecified abstraction dataset; not analyzed. |
| Clinical indication for antifungal prophylaxis | 0/13 | Not uniformly abstracted; prophylaxis agent at diagnosis is reported instead. |
| Diabetes mellitus | 0/13 | Not included as a structured variable in the prespecified abstraction dataset; not analyzed as a risk factor. |
| Iron overload/ferric overload | 0/13 | Not included as a structured variable; not analyzed. |
| Targeted/immunomodulatory agent exposure | 13/13 | Agent names were recorded when present; exact timing and indication were not uniformly captured. |
| Systemic corticosteroid dose at diagnosis | 13/13 | Prednisone-equivalent dose at diagnosis was abstracted. Duration was not uniformly captured. |
| Active GVHD at diagnosis | 13/13 | No missing values in prespecified abstraction dataset. |
| Pulmonary GVHD/BOS or structural lung factors | 13/13 | Pulmonary host-defense/structural variables were abstracted descriptively. |
| Antifungal prophylaxis at diagnosis | 13/13 | No missing values in prespecified abstraction dataset. |
| Azole TDM | 13/13 | Whether TDM was obtained, nearest level, timing, and interpretation were recorded when available. |
| BAL culture | 10/13 | Seven positive, three negative, and three not obtained/not recorded. |
| Sputum culture | 11/13 | Two positive, nine negative, and two not obtained/not recorded. |
| Molecular evidence | 13/13 | Field populated for all cases, but non-positive entries could not always be distinguished from not performed. |
| Amphotericin B formulation | 13/13 | Formulation was recorded; amphotericin dose was not captured. |
| End-of-treatment outcome | 0/13 | Not uniformly captured. |
| In-hospital mortality | 0/13 | Not uniformly captured. |
| 30-day, 42-day, and cumulative 90-day all-cause mortality | 13/13 | Derived from recorded death fields and dates when available. |

**Supplementary Table S3. Operational definitions used in the study dataset**

| **Derived variable** | **Operational definition** |
| --- | --- |
| Neutropenia at diagnosis | Absolute neutrophil count <500/µL at diagnosis. |
| Mold-active azole prophylaxis | Prophylaxis with posaconazole, voriconazole, itraconazole, or isavuconazole. |
| Mucorales-active prophylaxis | Prophylaxis with posaconazole or isavuconazole. No patient was receiving isavuconazole prophylaxis at diagnosis. |
| Disseminated mucormycosis | Involvement of at least two noncontiguous organs, or sterile-site involvement together with another organ site. |
| Diagnostic recognition to active therapy | Categorical field in dataset: <24 hours or >24 hours from diagnostic recognition to first active antifungal therapy. |
| Day-42 clinical response | Resolved = complete resolution; improved = partial improvement without complete resolution; worsened/death = clinical deterioration or death by day 42 as recorded in the clinical dataset. |
| Cumulative 90-day all-cause mortality | Death occurring by day 90 after diagnosis/onset, including deaths that occurred within the first 30 days. |
| Key diagnostic specimen/pathway | Summary field capturing the dominant route to diagnosis recorded in the dataset; analyzed separately from proven/probable EORTC/MSGERC adjudication. |

**Supplementary Table S4. Deidentified disease, transplant, and host context by case**

| **Case** | **Underlying disease** | **HSCT type** | **Donor type** | **Conditioning** | **Days from HSCT** | **Active GVHD** | **GVHD type** | **Pulmonary GVHD/BOS** | **Steroid dose mg/day** | **Neutropenia** | **Other targeted/immunomodulatory agent recorded** |
| --- | --- | --- | --- | --- | --- | --- | --- | --- | --- | --- | --- |
| 1 | CLL | Allo | MUD | Reduced | 1027 | Yes | Chronic | No | 10 | No | N/A |
| 2 | DLBCL | Auto | Self | Myeloablative | 1080 | No | None | No | 0 | Yes | Epcoritamab |
| 3 | CLL | Allo | MRD | Reduced | 1576 | Yes | Chronic | No | 0 | No | Pembrolizumab |
| 4 | T-cell lymphoma | Allo | cord blood / double cord | Reduced | 4063 | No | None | No | 0 | No | N/A |
| 5 | AML | Allo | MUD | Reduced | 25 | No | None | No | 0 | No | N/A |
| 6 | AML | Allo | MUD | Reduced | 41 | No | None | No | 0 | Yes | Abatacept |
| 7 | ALL (Ph-like B-cell ALL) | Allo | MUD | Myeloablative | 420 | No | None | No | 0 | No | N/A |
| 8 | CML | Allo | MUD | Myeloablative | 1328 | Yes | Chronic | Yes | 2.5 | No | N/A |
| 9 | AML | Allo | MUD | Reduced | 260 | No | None | No | 0 | No | Venetoclax |
| 10 | AML | Allo | MRD | Reduced | 192 | Yes | Acute | Yes | 60 | No | N/A |
| 11 | CML | Allo | MRD | Reduced | 934 | Yes | Chronic | Yes | 60 | No | N/A |
| 12 | MM | Auto | Self | Myeloablative | 663 | No | None | No | 0 | Yes | Daratumumab; pomalidomide; dexamethasone |
| 13 | VEXAS syndrome | Allo | MRD | Reduced | 681 | No | Chronic | Yes | 0 | Yes | rituximab approximately 1 week before diagnosis for EBV/PTLD concern; donor lymphocyte infusions approximately 4 to 5 months and 3 to 4 months before diagnosis |

**Supplementary Table S5. Deidentified diagnostic and microbiology details by case**

| **Case** | **Primary site** | **Secondary sites** | **Disseminated** | **EORTC/MSGERC class** | **Histopath invasion** | **Sterile-site culture** | **BAL culture** | **Sputum culture** | **Molecular evidence** | **Key pathway** | **Genus** | **Species** | **ID method** | **Co-pathogens** |
| --- | --- | --- | --- | --- | --- | --- | --- | --- | --- | --- | --- | --- | --- | --- |
| 1 | CNS | None | No | Proven | Yes | Y | N/A | N/A | N | Histopath | Rhizomucor | pusillus | Sequencing/PCR | None |
| 2 | Pulmonary | None | No | Probable | No | N | Y | N | N | BAL | Rhizopus | Not identified | Culture morphology | Aspergillus fumigatus complex |
| 3 | Pulmonary | None | No | Proven | Yes | Y | Y | N | N | BAL | Mucor | Not identified | Culture morphology | None |
| 4 | Cutaneous | None | No | Proven | Yes | Y - tissue (skin, left upper arm) | N/A | N/A | N | Histopathology plus tissue culture | Rhizopus | Not identified | Culture morphology | None |
| 5 | Pulmonary | CNS | Yes | Proven | Yes | Y - tissue, blood, pleural, CNS | N | N | Y - Karius | Molecular | Rhizomucor | pusillus | NR | None |
| 6 | ROS-Sinus | None | Yes | Proven | Yes | Y - tissue (oral cavity) | N | N | N | Histopathology plus tissue culture | Mucor | Not identified | Histopathology plus culture morphology | Enterococcus faecium, Stenotrophomonas maltophilia,Limosilactobacillus fermentum, Staphylococcus epidermidis |
| 7 | Pulmonary | Pleura/right hemithorax with diaphragmatic/abdominal extension | Yes | Proven | Yes | Y - thoracic tissue / pleural cavity empyema-right hemithorax tissue | Y | Y | N | Histopathology plus tissue culture | Rhizopus | Not identified | Histopathology plus culture morphology | Aspergillus, and Enterococcus faecalis; anaerobic gram-negative organisms / Bacteroides |
| 8 | ROS-Sinus + Pulm | None | No | Probable | No | N | Y | Y | N | BAL | Rhizopus | Not identified | Culture morphology | Pseudomonas aeruginosa; Enterobacter cloacae complex |
| 9 | ROS-Sinus | None | No | Proven | Yes | Y - Tissue | N | N | N | Histopathology plus tissue culture | Rhizopus | oryzae | Histopathology plus culture morphology | None |
| 10 | Pulmonary | None | No | Probable | No | N | Y | N | N | BAL | Rhizopus | Not identified | BAL smear plus culture morphology | Klebsiella oxytoca; Pseudomonas aeruginosa; human rhinovirus/enterovirus |
| 11 | Pulmonary | None | No | Proven | No | Y - lung tissue (right upper lobe) | Y | N | N | BAL + Histopath + Tissue | Rhizopus | Not identified | Culture morphology plus fungal smear | Enterococcus faecalis; Staphylococcus haemolyticus |
| 12 | ROS-Sinus | None | No | Proven | Yes | Y - Tissue | N/A | N | N | Histopath + Tissue culture | Mucor | Not identified | Histopathology | Aspergillus flavus |
| 13 | Pulmonary | None | No | Probable | No | N | Y | N | N | BAL | Mucor | Not identified | Culture morphology plus fungal smear | Stenotrophomonas maltophilia; Chryseobacterium arthrosphaerae; ESBL E. coli CRBSI; EBV DNA/possible EBV-associated LPD |

**Supplementary Table S6. Deidentified prophylaxis and azole therapeutic drug monitoring details by case**

| **Case** | **Prophylaxis drug** | **TDM obtained** | **Nearest level** | **Units** | **Days from diagnosis** | **Timing** | **Clinical interpretation** |
| --- | --- | --- | --- | --- | --- | --- | --- |
| 1 | Fluconazole | N | N/A | N/A | N/A | N/A | Not done |
| 2 | Fluconazole | Y | 736 | ng/mL | 12 | Post-dx | Therapeutic |
| 3 | Posaconazole | Y | 961 | ng/mL | 27 | Post-dx | Therapeutic |
| 4 | Itraconazole | Y | 2830 | ng/mL | 35 | Post-dx | Therapeutic |
| 5 | Posaconazole | Y | 575 | ng/mL | -1 | Within 30d pre-dx | Subtherapeutic |
| 6 | Posaconazole | Y | 733 | ng/mL | 4 | Post-dx | Subtherapeutic |
| 7 | Voriconazole | Y | 657 | ng/mL | 13 | Post-dx | On voriconazole prophylaxis; post-diagnosis posaconazole level 657 ng/mL after treatment changes. |
| 8 | Posaconazole | Y | 1120 | ng/mL | 0 | Within 30d pre-dx | Therapeutic |
| 9 | Posaconazole | Y | 1040 | ng/mL | 0 | Within 30d pre-dx | Therapeutic |
| 10 | Voriconazole | Y | 461 | ng/mL | -3 | Within 30d pre-dx | Low/subtherapeutic pre-diagnosis level (461 ng/mL). |
| 11 | Voriconazole | Y | 756 | ng/mL | 33 | Post-dx | Therapeutic |
| 12 | Voriconazole | N | N/A | N/A | N/A | N/A | N/A |
| 13 | Posaconazole | Y | 710 | ng/mL | -2 | Within 30d pre-dx | Near prophylaxis threshold but below treatment-level exposure per clinical documentation; dose increased. |

**Supplementary Table S7. Deidentified treatment, source control, critical care, and outcome details by case**

| **Case** | **Initial regimen** | **Ampho formulation** | **Time to active therapy** | **Source-control surgery** | **Days to surgery** | **ICU** | **Mechanical ventilation** | **Vasopressors** | **RRT** | **Day-42 response** | **30-day death** | **Cumulative 90-day death** | **Mucor-attributable death** |
| --- | --- | --- | --- | --- | --- | --- | --- | --- | --- | --- | --- | --- | --- |
| 1 | liposomal amphotericin B + Voriconazole | L-AmB | <24 hours | Y | 1 | No | No | No | No | Resolved | No | No | No |
| 2 | liposomal amphotericin B + Posaconazole | L-AmB | >24 hours | N | N/A | No | No | No | No | Improved | No | No | No |
| 3 | liposomal amphotericin B + Posaconazole | L-AmB | >24 hours | Y | 6 days | No | No | No | No | Resolved | No | No | No |
| 4 | Posaconazole | N/A | >24 hours | N | N/A | No | No | No | No | Resolved | No | No | No |
| 5 | liposomal amphotericin B + Posaconazole | L-AmB | <24 hours | N | N/A | Yes | Yes | Yes | No | Worsen-Death | Yes | Yes | Yes |
| 6 | liposomal amphotericin B + Posaconazole | L-AmB | <24 hours | N (biopsy/unable to source control) | 1 | Yes | Yes | No | No | Worsen-Death | Yes | Yes | Yes |
| 7 | liposomal amphotericin B + Posaconazole | L-AmB | <24 hours prior to confirmation due to high suspicion | Y | 6 | Yes | Yes | Yes | No | Worsen-Death | Yes | Yes | Yes |
| 8 | liposomal amphotericin B + Posaconazole | L-AmB | <24 hours | N | N/A | No | No | No | No | Resolved | No | No | No |
| 9 | liposomal amphotericin B + Posaconazole | L-AmB | <24 hours | Y | 0 | Yes | No | No | No | Improved | No | No | No |
| 10 | Liposomal amphotericin B + voriconazole | L-AmB | <24 hours | N | N/A | Yes | Yes | No | Yes | Worsen-Death | Yes | Yes | Yes |
| 11 | liposomal amphotericin B | L-AmB | <24 hours | Y | 6 | No | No | No | Yes | Improved | No | No | No |
| 12 | liposomal amphotericin B | L-AmB | <24 hours | Y | 3 | No | No | No | No | Improved | No | No | No |
| 13 | liposomal amphotericin B + Posaconazole | L-AmB | <24 | N | N/A | Yes | Yes | Yes | No | Worsen-Death | Yes | Yes | Yes |

**Supplementary Table S8. Descriptive mortality by disease extent, source control, and ICU requirement**

| **Subgroup** | **n** | **30-day all-cause deaths** | **42-day all-cause deaths** | **Mucormycosis-attributable deaths** |
| --- | --- | --- | --- | --- |
| Localized/non-disseminated disease | 10 | 2/10 | 2/10 | 2/10 |
| Disseminated disease | 3 | 3/3 | 3/3 | 3/3 |
| Definitive source-control surgery performed | 6 | 1/6 | 1/6 | 1/6 |
| No definitive source-control surgery | 7 | 4/7 | 4/7 | 4/7 |
| ICU admission within 14 days | 6 | 5/6 | 5/6 | 5/6 |
| No ICU admission within 14 days | 7 | 0/7 | 0/7 | 0/7 |

**Supplementary Table S9. Outcomes by prophylaxis/breakthrough category**

| **Category** | **n** | **Pre-dx TDM available** | **Pre-dx TDM interpretation** | **Pulmonary/mixed** | **Disseminated** | **ICU** | **30-day deaths** |
| --- | --- | --- | --- | --- | --- | --- | --- |
| Mucorales-active prophylaxis (posaconazole) | 6 | 4/6 | case 5: 575 ng/mL, Subtherapeutic; case 8: 1120 ng/mL, Therapeutic; case 9: 1040 ng/mL, Therapeutic; case 13: 710 ng/mL, Near prophylaxis threshold but below treatment-level exposure per clinical documentation; dose increased. | 4/6 | 2/6 | 4/6 | 3/6 |
| Other mold-active azole prophylaxis (voriconazole/itraconazole) | 5 | 1/5 | case 10: 461 ng/mL, Low/subtherapeutic pre-diagnosis level (461 ng/mL). | 3/5 | 1/5 | 2/5 | 2/5 |
| Non-mold-active prophylaxis (fluconazole) | 2 | 0/2 | None available pre-diagnosis | 1/2 | 0/2 | 0/2 | 0/2 |

**Supplementary Table S10. Targeted comparison of selected published cohorts**

| **Study/source** | **Years/scope** | **Population** | **n / cohort type** | **Median onset after HCT** | **Breakthrough/prophylaxis reporting** | **Pulmonary involvement** | **Disseminated disease** | **Mortality endpoint** | **Interpretive use** |
| --- | --- | --- | --- | --- | --- | --- | --- | --- | --- |
| Current Mayo series | 2008-2025; single center | Adult HSCT recipients with proven/probable mucormycosis | 13; ICD-screened case series with institutional denominator | 681 days (IQR 260-1080; range 25-4063) | Any systemic prophylaxis 13/13; mold-active azole 11/13; Mucorales-active prophylaxis 6/13; pre-dx TDM 5/13 | 8/13 pulmonary or mixed pulmonary | 3/13 | 30-day death 5/13; day-42 worsened/death 5/13 | Contemporary granular case-level series emphasizing late-onset, prophylaxis-associated, often non-neutropenic disease. |
| Xhaard et al. (4) | 2003-2008; French multicenter cohort | Allogeneic HSCT recipients with mucormycosis | 29; nationwide multicenter allo-HSCT cohort; prevalence 0.4% among participating allo-HSCT recipients | 225 days (range 0-2693) | Breakthrough infection 23/29 (79%); at diagnosis: voriconazole 11, itraconazole 4, posaconazole 3, L-AmB + caspofungin 2, caspofungin 1, caspofungin + voriconazole 1, fluconazole 1 | Lung 20/29 (68%); sinus 11/29 (38%); other 3/29 (10%) | 5/29 (17%) | Overall survival 34% at 3 months and 17% at 1 year; attributable mortality 59% | Most directly comparable historical allo-HSCT mucormycosis cohort; our median onset is later and our early mortality appears lower, with caution about time-point differences. |
| Miller et al. (14) | 2007-2017; 10 North American transplant centers | Adults with hematologic malignancy and/or HCT with proven/probable invasive mucormycosis | 64 total; 39 HCT recipients (95% allogeneic) | HCT recipients: 263 days (IQR 108-659) | Antifungal prophylaxis before diagnosis 58/64 (91%): voriconazole 27/58, fluconazole 11/58, echinocandin 9/58, posaconazole 9/58, amphotericin B 2/58 | Pulmonary primary site 19/64 (30%) | 12/64 (19%) | Total cohort: 30-day mortality 36%, 1-year 63%; HCT recipients: 30-day 33%, 1-year 62% | Contemporary multicenter HM/HCT comparator in the era of posaconazole/isavuconazole; supports high mortality despite newer antifungals. |
| Bao et al. (15) | 2010-2020; single center | Allogeneic HSCT recipients with pulmonary mucormycosis | 21 pulmonary mucormycosis cases | 96 days after allo-HSCT (IQR 25.5-229; range 1-1924) | Breakthrough infection 90.5%; agents listed at clinical suspicion included fluconazole 2, voriconazole 10, posaconazole 6, and echinocandins 13 | 21/21 pulmonary by design | 6/21 (28.6%) extrapulmonary/disseminated involvement | 28-day mortality 7/21 (33.3%); 1-year mortality 15/21 (71.4%) | Recent pulmonary allo-HSCT-focused comparator; earlier onset and higher 1-year mortality than our series; all extrapulmonary cases died within 1 year in that report. |
| Riches et al. (5) | 1995-2008; CIBMTR registry analysis | Allogeneic HCT recipients with non-Aspergillus mold infection within 1 year after HCT | 124 NAMI cases: 72 mucormycosis, 52 fusariosis; 11,856 controls | Mucormycosis median 75 days (range 1-360); all NAMI median 48 days | Prophylaxis for mucormycosis cases: fluconazole 29/72 (40%), voriconazole 8/72 (11%), itraconazole 5/72 (7%), posaconazole 1/72 (1%), amphotericin 14/72 (19%), echinocandin 1/72 (1%), other 1/72 (1%), none 13/72 (18%); dosing/duration unavailable | All NAMI: sinus/respiratory 55/124 (44%); mucormycosis-only site distribution not separable in table | All NAMI: 5/124 (4%); mucormycosis-only not separable | For NAMI: 100-day OS 27%; mucormycosis 100-day OS 29%; 1-year survival after mucormycosis approximately 15% | Large registry benchmark showing poor survival and limited prophylaxis granularity; less directly comparable because cases were limited to first year after HCT. |
| Kontoyiannis/TRANSNET (3) | 2001-2006; 22 US HSCT centers; prospective surveillance | HSCT recipients with proven/probable IFI | 983 IFIs among 875 HSCT recipients; zygomycosis 77/983 (8%) in surveillance cohort and 66/718 (9%) in incidence cohort | Zygomycosis median 135 days; 12 cases occurred after 1 year | Detailed prophylaxis/TDM not reported in overview | Site distribution for zygomycosis not reported in overview | NR in overview | 1-year survival for zygomycosis 28.0% | National prospective surveillance benchmark; supports that our cohort has later onset than typical historical HSCT zygomycosis. |
| Lanternier et al. (2) | Published 2012; narrative review | Organ and stem cell transplant recipients with mucormycosis | Review; includes 10 studies with >10 allo-HSCT mucormycosis cases | Review summarizes median reported times after HSCT of 135-225 days | Prophylaxis/breakthrough described qualitatively; review notes breakthrough cases on voriconazole and posaconazole and summarizes prior voriconazole exposure in selected cohorts | Review summarizes HSCT mucormycosis as >50% pulmonary and 20%-40% sinus | Review states at least 10% of HSCT cases are disseminated | Review states disease-related mortality in HSCT recipients is at least 75% across several studies | Useful broad transplant review and context source, not a primary comparator cohort. |
| Boutin et al. (6) | Systematic review; search through April 19, 2023 | High-risk hematologic disorders receiving voriconazole or posaconazole prophylaxis | 1076 breakthrough IFI cases; 1093 pathogens; HSCT 279/1076 (25.9%) | Not HCT-specific/NR | Posaconazole 619/1076; voriconazole 457/1076. Mucorales 219/1093 overall; 149/466 (32.0%) with voriconazole vs 70/627 (11.2%) with posaconazole. TDM reported in 90/1076 and subtherapeutic in 32/90 (36%). | All bIFI with site data: localized lung 309/679 (45.5%); among Mucorales mold cases with site data, pulmonary 42/105 (40%) | All bIFI 316/679 (46.5%); among Mucorales mold cases with site data, disseminated 30/105 (27%) | Infection-related mortality 117/337 (35%); not mucormycosis-only | Best comparator for breakthrough/TDM context; not an HSCT-only mucormycosis cohort. |
| Axell-House et al. (16) | 2000-2020; single cancer center | Patients with hematologic malignancy and/or HSCT with breakthrough mucormycosis on mold-active antifungals | 103 breakthrough mucormycosis cases; 50 allogeneic HSCT recipients | NR | Mucorales-active antifungals in 16 (isavuconazole 9, posaconazole 6, amphotericin B 1); other mold-active agents in 87 (voriconazole 52, echinocandin 22, itraconazole 8, echinocandin + voriconazole 5). Posaconazole levels were available for 4/6 posaconazole breakthrough cases, including one low level on suspension. | Sinopulmonary 65/103 (63%) | 19/103 (18%) | 42-day mortality from treatment initiation: 11/16 (69%) with Mucorales-active antifungals vs 34/87 (39%) with other mold-active agents; 42-day mortality from symptom onset: 10/16 (63%) vs 22/87 (25%) | Important context for the prognostic concern raised by breakthrough disease on Mucorales-active prophylaxis; not directly comparable because it includes HM and HSCT patients and focuses only on breakthrough cases. |
| Imhof et al. (11) | 1998-2003; single center | Stem cell transplant recipients receiving voriconazole for treatment, prophylaxis, or empirical therapy | 13 breakthrough IFIs among 139 voriconazole-exposed patients; zygomycetes 6/13 | All breakthrough IFIs median day 180 after SCT (range 11-949); zygomycete-only median NR | All 13 were receiving voriconazole at diagnosis; median voriconazole exposure 47 days before breakthrough infection; all had prior azole prophylaxis | Zygomycetes: lung 5/6; GI tract 1/6 | Zygomycete-only disseminated disease NR | Overall: 9/13 died; zygomycete case table includes 4/6 deaths, one attributed to relapse | Early voriconazole-breakthrough signal; not directly comparable because voriconazole use included treatment/empirical indications, not only prophylaxis. |
| Siwek et al. (12) | 2003-2004; single center | HSCT recipients receiving voriconazole prophylaxis | 4 invasive zygomycosis cases among 45 HSCT recipients after voriconazole prophylaxis began; no cases in prior 3 years | NR; cases occurred on post-HCT days 27, 70, 68, and 187 in case narratives/autopsy timing | All 4 occurred during voriconazole prophylaxis and immunosuppression for presumed GVHD | Lung involvement in all 4; extrapulmonary involvement in at least 2 by autopsy | At least 2/4 with multiorgan/extrapulmonary involvement | All 4 died | Small early warning series highlighting vulnerability during voriconazole prophylaxis and GVHD immunosuppression. |
| Trifilio et al. (13) | 2002-2005; 13 cancer centers | Patients with hematologic malignancy or HSCT who developed zygomycosis after voriconazole exposure | 58 cancer patients; 56 hematologic malignancy; about 35 HSCT recipients (34 allo, 1 auto) | Among HSCT recipients: mean 150 days, median 98 days, range 11-925 | Voriconazole for primary/secondary prophylaxis in 92% and empirical therapy in 8%; mean exposure 80 days, median 50 days before zygomycosis | Lung 35/58 (62%); sinopulmonary 12/58 (20%); other 11/58 (18%) | NR as a separate category | Overall mortality reported as 73% | Large early multicenter voriconazole-associated zygomycosis series; useful for breakthrough context but population includes non-HSCT intensive chemotherapy patients. |

Note: This targeted comparison was included to contextualize the present series and was not designed or conducted as a systematic review or meta-analysis. Values were abstracted only when directly reported in the cited papers or their supplements. NR indicates not reported or not separable for the relevant population.

Abbreviations: bIFI, breakthrough invasive fungal infection; HCT, hematopoietic cell transplantation; HSCT, hematopoietic stem cell transplantation; L-AmB, liposomal amphotericin B; NAMI, non-Aspergillus mold infection; NR, not reported; TDM, therapeutic drug monitoring.
